# Reaching out to men in ending intimate partner violence: a qualitative study among male civil servants in Ibadan, Nigeria

**DOI:** 10.1080/17482631.2022.2128263

**Published:** 2022-10-18

**Authors:** Adebola Afolake Adejimi, Oluwaseun O. Akinyemi, Olutoyin O. Sekoni, Olufunmilayo I. Fawole

**Affiliations:** a Department of Community Health and Primary Care, College of Medicine, University of Lagos/Lagos University Teaching Hospital, Lagos, Nigeria; b Department of Community Medicine, University of Ibadan/University College Hospital, Ibadan, Nigeria; c Department of Health Policy and Management, University of Ibadan/University College Hospital, Ibadan, Nigeria; d Department of Epidemiology and Medical Statistics, University of Ibadan/University College Hospital, Ibadan, Nigeria

**Keywords:** Intimate partner violence, civil servants, men, perceptions, focus group discussion, Nigeria

## Abstract

Intimate Partner Violence (IPV) is one of the most pervasive human rights violation which has negative impacts on the health and well-being of individuals, families and communities.

**Purpose:**

The aim of this study was to explore the perceptions of male civil servants in Ibadan, Nigeria about the perpetration of IPV and to document their suggested measures to prevent IPV in our communities in Nigeria.

**Methods:**

Four focus group discussions were conducted among 36 male civil servants selected from Oyo State Secretariat, Ibadan using purposive sampling technique. Data were transcribed and analysed using thematic approach.

**Results:**

Six major themes were identified; awareness of the forms of IPV, women and men as victims, causes, attitude, consequences as well as the suggested strategies for the prevention of IPV. Physical and psychological abuse were mentioned across the groups. The respondents pointed out that women experience IPV more than men, but that men also experience it. Some respondents stated that physical and psychological abuse against female intimate partners were acceptable in some circumstances according to the societal norms. The negative effects of IPV on physical, mental and social well-being of the individual, families and society were mentioned. Suggested ways of preventing IPV include tolerance and patience which will promote healthy, respectful and non-violent relationships among intimate partners.

**Conclusion:**

Considering the perceptions and attitudes of these men to IPV, it is important to reach out to both genders for appropriate preventive and educational intervention in ending IPV among women and men.

## Introduction

Intimate Partner Violence (IPV) is one of the most pervasive human rights violation. It is one of the common forms of violence against women which is performed by a husband or male partner (World Health Organization, 2013). It occurs across social, economic, religious and cultural groups (Ellsberg & Heise, 2005). It is an act, behaviour or attitude which results in, or is likely to result in physical, sexual or psychological harm or suffering (Ellsberg et al., 2008). It includes acts of physical aggression such as slapping, hitting, kicking or beating; psychological abuse such as intimidation, constant belittling or humiliation; forced sexual intercourse and other forms of sexual coercion or any other controlling behaviour such as isolating a partner from family and friends, monitoring a partner’s movement or activities and restricting access to information or assistance (Krug, Mercy et al., 2002; World Health Organization, 2013). The nature of a violent act includes those acts that result from a power relationship, including threats and intimidation (Krug, Dahlberg et al., 2002). IPV devastates lives, fractures communities and stalls development (Devries et al., 2013; World Health Organization, 2013). It has a serious impact on women’s health and well-being and its cost to individuals, health systems and society in general is enormous (C Garcia-Moreno et al., 2005). IPV impoverishes individuals, families and communities, reducing economic development of the nation (Babu & Kusuma, 2016; Claudia Garcia-Moreno & Amin, 2016). It is a major cause of disability and death with the negative consequences of IPV affecting overall health of the victims and the welfare of their children (World Health Organization, 2009).

IPV is a complex social issue deeply rooted in the interaction of social, cultural, political, economic and biological factors (Dahlberg & Butchart, 2005). Social, cultural and religious beliefs influence the perceptions, prevalence, and manifestations of IPV across societies (Akinsulure-Smith et al., 2013). In some traditional societies including Nigeria, wife beating is largely regarded as a consequence of a man’s right to inflict physical punishment on his wife (Gage & Thomas, 2017; Krug, Dahlberg et al., 2002; Linos et al., 2013). Cultural justifications for violence usually follow from traditional notions of the proper roles of men and women. Societies often distinguish between “just” and “unjust” reasons for abuse and between “acceptable” and “unacceptable” levels of violence (Rani et al., 2004). In this way, certain individuals, usually husbands or older family members, are given the right to punish a woman physically for certain transgressions. Research from various settings have suggested that rates of violence by an intimate partner may be higher in settings where the behaviour is seen as normal and where it is believed that marriage grants men unconditional dominance and sexual access to their wives (Rani et al., 2004). In Nigeria, permissive social norms, where the husband have the right to beat his wife, appeared to significantly increase the odds of spousal violence at the state level (Linos et al., 2013) and reversed the protective effect of higher status on the likelihood of experiencing IPV by women (Benebo et al., 2018). Nigerian men would readily go with the norms that expect them to be in control of their home affairs and discipline their partners when needed (Benebo et al., 2018).

Although men can be victims of this type of violence, the majority of IPV globally is perpetrated by men against women and women suffer disproportionately from IPV, especially in terms of injuries (Caldwell et al., 2012; Carmo et al., 2011). A World Health Organization (WHO) multi-country study in 10 developing countries found that between 15% and 71% of women reported physical and/or sexual violence perpetrated by intimate partners at some point in their lives (C Garcia-Moreno et al., 2005). About 36% of women in urban sub-Saharan Africa experienced at least one form of IPV (Izugbara et al., 2020). The prevalence of physical IPV was 31% among South African women (Gass et al., 2011). In Tanzania, 27% of women experienced physical and/or sexual IPV in the past year (Kapiga et al., 2017). About 34% of Ghanaian women experienced IPV in the past year, with 21.4% reporting sexual and/or physical forms and 24.6% reporting emotional abuse (Alangea et al., 2018). One in four women in Nigeria reported having ever experienced IPV (Benebo et al., 2018). In a study among the male civil servant in Ibadan, Nigeria, 31.2% of the men reported carrying out at least one episode of psychological abuse, 23% reported sexual abuse and 11.7% reported physical violence against their intimate partners in the past year, while about 30.7% of them supported wife beating in certain circumstances (Adejimi et al., 2014).

IPV has adverse effects on health and well-being of the female victims which can last a lifetime (Antai, 2011; Campbell et al., 2002; Sanz-Barbero et al., 2019). These include injuries, chronic pain, physical disability, sexual and reproductive health complications, including sexually transmitted infections and mental illnesses such as post-traumatic stress disorder, depression, substance abuse and suicide attempts (Campbell et al., 2002; Decker et al., 2014; Ellsberg & Heise, 2005; Krug, Dahlberg et al., 2002). Higher prevalence of chronic disease was observed in abused women than in never abused women in Spain (Ruiz-Pérez et al., 2007). Violence is also a recognized risk factor for acquiring HIV/AIDS, especially for women in Africa through limited negotiation of safer sexual practices and increased sexual risk-taking behaviour (Campbell et al., 2008; Patrikar et al., 2017; World Health Organization & UNAIDS, 2004).

Effective interventions can be developed when qualitative studies that explore the socio-cultural context of affected population and how they interpret phenomena (Jewkes et al., 2015). Qualitative research methods are important in public health research in order to provide the baseline information and to find the meaning behind the numbers. The use of qualitative research method in this study provides important insight and understanding of the context, meanings, beliefs and values associated with IPV. Previous studies including those from Nigeria have focused on this problem from the females’ perspective. Less work has been done to investigate the factors influencing men’s motivations and reasons of perpetrating violence against intimate partners. Such work is needed to inform development of evidence-based public health strategies to reduce men’s use of such violence. It is important to encourage men to be involved in the process of ending IPV by giving them the opportunity to suggest the ways out of this public health problem. Men play many roles in the society as fathers, husbands, uncles, religious leader, professionals, policymakers and local and national leaders. They are part of the group that can bring a change in societal norms. Focusing on male civil servants in Nigeria has implications on how IPV is addressed from the perspective of these government employees. They can be involved in policy making which can bring a change in societal norms and standards. The aim of this study was to explore the perceptions of male civil servants in Ibadan, Nigeria about the perpetration of IPV and to document their suggested measures to prevent it in their communities.

## Methods

This article reports the findings of the qualitative component of mixed-methods research on IPV among men in Ibadan, Nigeria. The details of the study design have been previously described (Adejimi et al., 2014). Focus group discussions (FGD) were conducted to provide formative data for the development of a survey instrument for the quantitative study. This technique involves a number of participants discussing a predetermined topic in a session led by one or two moderators supported by a question guide. The discussion between participants relies on the moderator’s skills which encourage participants to talk freely (Puchta & Potter, 2004). In this study, our discussion consisted of open-ended questions about the participants’ view on IPV perpetration in their communities and suggestions on the preventive strategies. Participants were able to express their own thoughts and feelings among others in a non-threatening and informal environment. Some participants felt comfortable to give their personal experience about IPV perpetration. The empirical data derived from interviews with the male civil servants were used to describe the subjective and unique meanings of violent behaviour in intimate relationships of men as perceived by them.

### Study area

This study was carried out in Ibadan, the ancient capital city of Oyo State, located in south-west Nigeria. Ibadan has a population of about 3 million (National Population Commission, 2006). Majority of the people living in the urban area are civil servants, while those in the peri-urban and rural areas are mainly farmers, artisans and petty traders. The inhabitants are mainly Yorubas.

### Study site

The study was conducted in Oyo State Secretariat which is situated in Agodi, Ibadan North Local Government Area of Ibadan. There are 15 ministries located in the secretariat with an estimate of 6,075 staff of which 3,362 and 2,713 are junior and senior staff, respectively. The male-to-female ratio of workers is approximately ratio 1:1.

### Selection of participants

A purposive sampling technique (Robson, 2011) was used for selecting participants who were recommended by the Oyo State Ministry of Health for the FGDs. The FGD participants were selected male civil servants working in the different Ministries in the Oyo State Secretariat. Their selection was stratified by grade level and the consisted of senior staff on grade seven and above and junior staff on grade six and below. The participants were recruited for the FGD with the assistance of the social workers at the State Ministry of Health, Oyo State Secretariat in Ibadan. The participants were invited after the study was explained to them and they were willing to participate. A total of 36 men participated in four FGDs conducted. Two FGDs were conducted among the senior staff; the majority of the senior staff were married. The junior staff were stratified by marital status and thus one FGD was conducted among the married junior staff and another one among the single junior staff to ensure homogeneity based on the assumption that married and single men may have different opinions on IPV. It was also necessary to have separate FGD among the senior and junior staff so that participant could be free to discuss the topic. It was recommended that 6–12 persons are ideal for a focus group discussion (Robson, 2011); in this study, each group consisted of between 8 and 10 participants.

### Data collection

The FGDs were conducted over a 2-week period at the seminar room of the Ministry of Health Oyo State Secretariat, Ibadan which is a private room. Only the participant and research team members were present during the discussions. A focus group discussion guide was used to obtain information on the participants’ view and perception about IPV, types and causes of IPV, consequences of IPV and the suggested ways of preventing IPV. The guide comprised of open-ended questions and suitable probes was designed by the research team members. The discussions were facilitated by the authors who had public health background as well as training in qualitative research methods. The groups were moderated by the first two authors who focused on being good listeners and non-judgemental. They encouraged the participants to share their experience while capturing the essence of the discussion. The authors took turns in moderating all the discussions and acted as observers when they were not facilitating a discussion. The moderator discussed issue of confidentiality with all the participants before each discussion, obtained verbal informed consents and ensured that the atmosphere was conducive for the discussion. The participants were assured of the confidentiality and anonymity of the information provided during the discussions and that their employer will not have access to the information provided.

The sessions were audio-taped after the moderator had obtained permission from the participants. A designated note taker took detailed notes to assist in the transcription process. The FGDs were conducted in English Language and participants were allowed to use the local language (Yoruba) to express their views when necessary. All participants were asked, one at time, to talk about their views on the perpetration and experiences of IPV. To deepen the conversation, we posed questions such as “could you please explain what you meant by … ?” Participants were encouraged to present new perspectives and themes in the sessions. Initially, some participants were enthusiastic about the discussion and talked freely, while others were cautious; but after a while, all the participants spoke freely. Each discussion lasted for about 60 to 90 minutes. At the end of each FGD, questions were asked about the experience from their participation in the group discussion, and participants were encouraged to contact the authors afterwards if they had any further questions. The team met to debrief the emerging themes after each FGD and saturation was achieved after the four FGDs. The discussions were audio-recorded and kept in a password-locked computer that was accessible to the members of research team only.

### Data analysis

Using thematic analysis (Green & Thorogood, 2009), the authors reviewed the data set carefully by listening to the recordings repeatedly. Grounded theory approach and automated coding were used for the data analysis. The field notes were studied in order to understand the meaning of the messages. This was followed by transcribing the audio recordings verbatim starting with the most interesting interviews. The English transcripts for each of the audio recordings were further evaluated to ensure translation accuracy and were imported into NVIVO version 8 for qualitative data analysis. Interesting features in the transcripts and field notes were identified, sorted and coded; and the codes were later arranged systematically. Thereafter, the identified codes were grouped; and from these groups, the themes were identified. After deciding on the dominant themes, the final list of themes was reported and the data were arranged into the respective themes.

### Ethical considerations

This study was part of a larger study which had received approval by the Oyo State Research Ethics Review Committee with reference number AD 13/479/128. Participants were informed of their rights to decline or withdraw from the study at any time without any adverse consequences on themselves or their work. A pamphlet introducing the research team members and describing the nature and the procedures of the study, as well as the risks, benefits and the rights associated with participating in the study was read to the participants. Verbal informed consent was obtained from each participant individually after explaining the purpose of the study to them. Participants were assured of confidentiality and anonymity of the data collected. They were also assured that their employer will not have access to the information provided during the discussions.

## Results

### Participants’ characteristics

A total of 36 male civil servants participated in the four focus groups. (Table I) All the participants were from the Yoruba tribe, which is the dominant tribe in the south-western Nigeria. The findings from the FGDs are grouped based on similar themes: respondents’ views about the meaning and how common IPV is in our society, awareness of types of IPV, causes and factors responsible for IPV, attitude to IPV, possible consequences of IPV and suggested strategies for the prevention of IPV. (Table 2) Some quotes from the participants’ discussion are used to illustrate the themes identified.

**Table I.** Characteristics of focus group discussion participants

Focus group	Work category	Number of participants	Age range (years)	Marital status
1	Senior civil servants	8	37 - 52	Married
2	Junior civil servants	10	27 - 40	Married
3	Senior civil servants	8	30 - 42	Married
4	Senior civil servants	10	22 - 32	Single

**Table 2.** Overview of themes and subthemes.

Themes	Subthemes
Views on the understanding of IPV	Disagreement Misunderstanding Crisis & Aggressive use of force (between intimate partners in marital, courtship or dating relationship).
Awareness of the forms of IPV	Physical violence Sexual violence Psychological/ emotional abuse Not sure that controlling behaviour is a form of violence Atypical form of violence (“Spiritual violence”)
Causes of IPV	Illiteracy Poverty and poor living conditions Natural tendencies of men Negligence of duties and roles in relationships Lack of respect and non-submission Infidelity Interference from the in-laws Westernization and gender equality
Attitude to IPV	Physical abuse is not justifiable Sexual violence and controlling behaviour can be justified Psychological abuse is better that physical violence
Possible consequences of IPV	Negatively affects the victims, perpetrator, children & society Physical injuries Mental health problems Social problems
Suggested preventive measures against IPV	Spirituality Tolerance Patience Submission Counselling Poverty alleviation Better housing Job creation for women

### Theme 1: respondents’ views about the meaning of and how common IPV is

Respondents across the groups gave their views about the meaning of IPV in similar ways. They perceived IPV as a form of disagreement, misunderstanding, crisis, or aggressive use of force between intimate partners who are either in marital, courtship or dating relationships. Some senior civil servant gave their opinions as shown below:

*It is a crisis that usually occurs between two people that live together as husband and wife, they may not necessarily be married legally but in as much as they are living together, share feelings and even have children, they are intimate partners*. (FGD 1: M, SCS, 52 years)

*It is maltreatment in the context of any relationship. The word ‘intimate’ means strong friendship and ‘violence’ means aggressive use of force. Intimate partner violence means aggressive use of force to cause harm to your partner or forcing somebody to do something against her will*. (FGD 3: M, SCS, 35 years)

Many respondents across the groups agreed that it is a common problem in our society as conflict is inevitable in human relationships. They were of the opinion that it is a common problem that is peculiar to human beings and that is common even among the educated, but much more common among the illiterates who do not have formal education. Some were of the opinion that although it is a common problem, it does not cut across all the societies in the world. Here are some of the opinions that they shared:

*Yes, it is a common problem and it should be a common problem because individuals’ views and nature differ. Occasionally, there could be instance of violence. At times it may not be intentional. It is partly common among the educated ones. It is mostly common among illiterates because they cannot understand each other*. (FGD 1: M, SCS, 40 years)

*It is a common problem that has been for ages. … . It may not be common in the developed world but in our society, it is very common*. (FGD 2: M, JCS, 29 years)

*It is common but it does not cut across all sectors of the society depending on the background of the individuals involved*. (FGD 3: M, SCS, 33 years)

### Theme 2: awareness of types of IPV

All the discussants across the groups mentioned and agreed that physical violence, sexual violence and psychological/emotional abuse are recognized forms of violence in intimate relationships. Respondents described physical violence to include exchanging of blows, beating/battering, slapping, use of weapon and pouring of acid while sexual violence may include forcing a partner to have sexual intercourse. With regard to psychological/emotional abuse, in the opinion of the discussants, this entails the use of abusive or insulting words or songs against one’s partner, withdrawal from normal responsibility towards a partner or rejecting a woman’ food. They described the forms of IPV as follows:

*Some are physical and some are psychological. The physical one is exchanging of blows, fighting e.t.c. The other one is keeping quiet. If my wife does something wrong and I fail to utter a word, she might not be able to sleep that night*. (FGD 1: M, SCS, 45 years)

*It is not only by fighting or quarreling that a woman can be punished. You can reject her food. Doing this is more painful than beating. If I do this to my wife for 2-3 days she will kneel down and be crying*. (FGD 3: M, SCS, 38 years)

*The most common type is the use of abusive words against each other*. (FGD 4: S, JCS, 32 years)

Only a few of the participants mentioned controlling behaviour (such as denying ones partner access to relatives and expecting ones partner to take permission before engaging in certain activities) as a form of violence. Majority of the respondents did not agree that controlling behaviour is a form of violence in intimate relationships. Many of the discussants were of the opinion that it is the husbands’ right to control his partner in relationships. They said it is the right of a man to control the woman and that is socially acceptable. They gave their views as follows:

*If necessary, it is acceptable to deny her access to some certain things such as money and people, for example, deny her access to visit her relatives or even access to her friends*. (FGD 2: M, JCS, 35 years)

*It is not violence [for a husband to control the wife] because as a master in the house, the husband is the sole provider of peace so anything he sees that will cause problem or tarnish the image of the family must be monitored*. (FGD 3: M, SCS, 36 years)

While some discussants agreed that there can be sexual violence in relationships especially among the unmarried, some of the discussants did not agree that a wife has a right to refuse sexual intercourse whenever the husband wants it. These are some of their opinions:

*We see in some western magazine reports of women who suffer from sexual abuse but do you think an African woman would come out openly to say her husband raped her? So, there is nothing like rape in marital relationship. If such a case should occur and a woman should accuse her husband of raping her, she will be asked to explain what her role is in the house other than pleasing her husband*. (FGD 3: M, SCS, 39 years)

*In terms of boyfriend and girlfriend it is an offence, but between married couples, it is not because once you are married you are expected to be ready anytime your husband makes the request except you are menstruating. Because if a woman denies a man his right, he can go out to do it outside and if he does not want to go outside, he will have to force the woman*. (FGD 4: S, JCS, 30 years)

Interestingly, some discussants mentioned that there is also an atypical form of violence which can be referred to as “spiritual violence” in intimate relationship whereby a man put “magun” (which literality means “do not climb”) on the partner if he suspects her of unfaithfulness. It was agreed that this can harm the partner if she is not able to have sexual intercourse with a man for certain number of days. Some responded that:

*Spiritual violence: when a man suspects his wife to be unfaithful, he can put ‘magun’ (thunderbolt) on her … If she doesn’t meets the unfaithful sexual partners within the specified days, then the “magun” will affect her negatively; but if she does, the unfaithful sexual partners will be negatively affected*. (FGD 2: M, JCS, 34 years)

### Theme 3: causes and factors responsible for IPV

A number of factors were highlighted by the discussants as causes of IPV in relationship. Poverty and poor economic situation of the family was mentioned in all the groups. Married respondents were of the opinion that inability to provide for the family, for example, inability to pay children’s school fees and providing for house expenses, is a major factor influencing the occurrence of IPV. Some respondents were of the opinion that men have natural tendency to perpetrate violence because of their hormone “androgen”. It was also mentioned that men have absolute power and control in relationship, thus when women disagree with their decisions, violence could ensue. Negligence of normal roles for example, negligence on child care, refusal to prepare food, unfaithfulness, lack of respect for the husband or the in-laws and sexual denial are some other factors that can cause IPV. Some respondents were of the opinion that western civilization and education has led to lack of submission of women to their husband and thus women empowerment and gender equality are other factors that has contributed to the occurrence of IPV in relationships. Some of their responses are as follows:

*The African tradition gives men the privilege of control and power over their wives. In a male-female relationship, you will see traces of use of force to achieve that control*. (FGD 1: M, SCS, 48 years)

*For every man there is a sort of androgen, I mean hormones-natural tendencies … .when the partners disagree, this androgen will rise and lead to violence. If I expect my wife to take care of my children and she pays little or no attention, violence might be triggered off in the household*. (FGD 2: M, JCS, 40 years)

*Civilization: men want total submission from their wives but civilization has changed most women’s views about being submissive. Thus, the issues of equality can to lead to violence*. (FGD 2: M, JCS, 32 years)

*Women empowerment programme: this programme is meant to train women to be industrious but some have been using that as a means to advocate for equal rights. Empowerment should not be misunderstood for equal right*. (FGD 3: M, SCS, 35 years)

Poor economic status, poor living conditions, having an illiterate partner, transfer of aggression, cultural differences, societal norms, polygamy and use of alcohol were some of the causes of IPV that were mentioned by some of the respondents. Some of their views are shown below:

*The economic situation is a reason … The husband may not be able to meet up with his responsibilities and the wife’s contribution too may not be up to expectations. Transferred aggression may result from this situation*. (FGD 2: M, JCS, 30 years)

*We men do smoke and drink and the after effect of this can cause violence and this can lead to disagreement in the house*. (FGD 4: S, JCS, 28 years)

*Where we come from also matters … intertribal marriage can lead to violence between a man and his wife. What is forbidden in one society might be allowed in another society*. (FGD 3: M, JCS, 31 years)

*Sexual denial or sexual incompatibility can cause violence*. (FGD 1: M, JCS, 40 years)

### Theme 4: attitude to IPV

Majority of the respondents were of the opinion that physical violence against intimate partners in any form is not justifiable or acceptable in any circumstance. Some respondents tried to justify sexual violence in marital relationship as the right of a man to use any means to have a sexual intercourse with the wife even if she is not interested because it is better for him to do that than to have extra-marital affairs. Some respondents were of the opinion that psychological/emotional abuse is better for correcting their partners than physical violence. Majority of the respondent agreed that controlling behaviour is justifiable in relationship as the social and cultural beliefs supports male dominance. Some of their responses are as follows:

*It is abnormal for a right thinking man to beat his wife and the same apply to a right thinking woman, she should not use weapon to fight her husband*. (FGD 1: M, SCS, 37 years)

*Physical violence is not justifiable but psychological violence is justifiable and acceptable. In marital relationship, there is an extent to which it sexual violence is justifiable*. (FGD 1: M, SCS, 42 years)

*I believe that controlling my wife’s behaviour is justified and acceptable because if we compare the lifestyle of a woman living alone and that of a woman living with her husband you will see a difference. As far as men are concerned there should be limit to a woman’s freedom*. (FGD 2: M, JCS, 38 years)

### Theme 5: possible consequences of IPV

Various effects of IPV were mentioned across the groups. These include bruises, blindness, body injuries and fractures. The participants agreed that IPV will affect the perpetrator and the victim negatively. Hypertension, mental health problem such as depression, sleeping problems and anxiety were also mentioned as the possible consequences of IPV. Effects of IPV on social health that were mentioned include divorce and loss of social respect especially for the woman. Some discussants mentioned suicide and death as health effect of IPV. Some of the responses were as follows:

*It can also be serious to the point that one of them will lose his/her life. When physical violence between intimate partners, there is a tendency that someone gets wounded on a delicate part of the body*. (FGD 1: M, JCS, 34 years)

*In my place, a woman who quarrelled with her husband and packed out of the house will lose her respect in the society; she will not be able to pack to her father’s house. It is a man’s world; a man can do anything and get away with it but a woman will be despised*. (FGD 2: M, JCS, 40 years)

Discussants agreed across the groups that IPV has a negative effect on the children and the family. They mentioned that these children can become violent with their peers; and they can become violent in their homes and in the society later in life. It can also make children develop resentment towards the parent they see as the perpetrator of IPV. Also, there can be family disintegration which may lead to broken homes and divorce and these can negatively affect the children’s development and educational attainment.

*The boys might grow up to be abusing their wives while the girls might be scared of entering a relationship*. (FGD 2: M, JCS, 27 years)

*Violence affects the children’s education and the children will start exhibiting violent behaviors in the neighborhood. Children will start showing bad habit like smoking and taking hard drugs*. (FGD 3: M, SCS, 40 years)

As a family is a unit of every society, discussants agreed that violence in a family will affect the society in general. The effects of IPV on work were also mentioned as loss of concentration at work, transfer of aggression to co-workers and reduced productivity. Some of their responses are highlighted as follows:

*The family is the smallest unit in the society. So once the family is affected, the society will also be affected*. (FGD 4: S, JCS, 24 years)

*There will always be a reflection of intimate partner violence you experience at home in your place of work and you will lack concentration. This will also lead to low productivity at work. You may also transfer the aggression to other co-workers. You will lose concentration in whatever you are doing at work and you may transfer the aggression to other members of staff*. (FGD 1: M, SCS, 37 years)

### Theme 6: prevention of IPV

Participants felt that it is the responsibility of individuals, societies and governments to prevent IPV. It was suggested that partners in relationships should be pious, matured, tolerant and patient. They should also respect each other, love one another and communicate with each other regularly. They should have courtship before marriage and should avoid involving a third party in their relationships. Women should not misunderstand their empowerment to mean liberation; they should be submissive to their husbands.

*The wife must avoid hearsay and shun bad associations. Women who have attained higher educational status should not be too arrogant. They should be submissive and understand their roles in the family*. (FGD 3: M, SCS, 39 years)

Participants also recommended that religious leaders should encourage pre-nuptial and post-nuptial marriage counselling. They recommended that the traditional norms that promote violence should be changed but that traditional marriage should still be encouraged. They also suggested that in-laws should avoid interfering with married couples.

*In the olden days, religious leaders preach about how to strengthen family life but nowadays the preaching is concentrated on wealth and prosperity. Religious leaders should preach about non-violent relationship between husband and wife*. (FGD 3: M, SCS, 39 years)

*In-laws should avoid disturbing married couples, violence between intimate partners are sometimes instigated by in-laws*. (FGD 2: M, JCS, 37 years)

Participants recommended that government at all levels should provide conducive environment for families to live and should enlighten the public on IPV.

*Government need to organize programmes or seminars and introduce some educative programmes on television, radio or papers for people to get the latest information on things that are happening around especially about this violence. This will enlighten couples on how to have a happy family life*. (FGD 1: M, SCS, 41 years)

*The government can provide employment opportunities and can organize self-assisted project such as fashion designing, craftwork e.t.c. I mean job creation for our women: if a woman is working, financial burden will not be on the man only and this will reduce this violence*. (FGD 2: M, JCS, 36 years)

## Discussion

We collected qualitative data on views of men in the civil service about their understanding of IPV, risk factors and possible solutions for this problem. In this study, men seem to have good understanding of IPV, going by the views they expressed. In a study conducted among married men in urban communities of Ibadan, Nigeria, 55.6% of respondents had good knowledge about IPV (O. I. O. I. Fawole et al., 2010). In the study, good knowledge was associated with younger age group, monogamous unions, higher educational attainment and non-use of alcohol. Our study participants alluded that IPV is a common phenomenon in the Nigerian society which has also been reported by researchers globally (Abramsky et al., 2011; Ackerson et al., 2008; Okenwa-Emegwa et al., 2016).

While the participants described a plethora of IPV forms, many did not see controlling behaviour as a form of violence. Grounding their assertion in Nigeria’s patriarchal culture, they argued that it was the man’s responsibility to lead and control his wife. However, research has shown that controlling behaviour, where someone’s movement is restricted, isolated from family and friends or given reduced access to family resources, is a form of IPV (World Health Organization, 2012). An unusual type of violence identified is “spiritual violence” which entails the use of charms or *juju*, usually by a jealous husband to ensure his partner’s fidelity. Although not referred to as “spiritual violence”, the use of *magun* to penalize women who engage in adultery in the traditional Yoruba setting has been well described in literature (Kehinde, 2014; Ojo, 2013). This might, arguably, be an extension of male dominance and control in these settings, especially since there is no equivalent means to check adultery in men. Also, views about “control” over women and male dominance as expressed by many of the participants, has been identified to be important drivers of violence against women from their spouses and intimate partners, not only in developing countries but globally (Jewkes, 2002; Lelaurain et al., 2018; Ozaki & Otis, 2017; World Health Organization, 2012). Patriarchy has continued to make the journey towards gender equality, with the anticipated reduction or obliteration of violence against women and girls, a difficult task (Carter, 2015; Tonsing & Tonsing, 2019).

Understanding the severity of violence is important and it requires the awareness of the kind and level of violence as well as the context in which it occurs. The participants in this study did not show adequate level of understanding of the severity of violence and the co-occurrence of different forms of violence. There is a big difference in remaining silent and perpetrating severe physical abuse against intimate partner. This has implications on women’s health. Research findings show that co-occurrence of different forms of violence is common and is the norm (Adejimi et al., 2022; Hamby & Grych, 2012). Women experiencing severe combined physical, emotional and sexual abuse have poorer quality of life and mental health than women experiencing other forms of abuse (Hegarty et al., 2013). In addition, participants’ understanding of what actually constitutes as IPV is also inadequate. Participants recognized some key forms of abuse, particularly as it pertains to control within intimate relationship. It is interesting that men considered remaining silent or rejecting food as forms of IPV but that control is not. Remaining silent or rejecting food could be forms of passive resistance which may be considered as non-violent behaviour. Educational intervention directed at men to prevent IPV perpetration should address the areas of inadequate understanding of IPV.

Furthermore, respondents were of the opinion that lack of formal education was a major predisposing factor for IPV. In a WHO multi-country study conducted in 10 countries including three from Africa, possession of at least a secondary school education was found to be protective from IPV (Abramsky et al., 2011). Just as the findings of this study showed that lack or low level of formal education predispose women to IPV, other literature indicated that achieving secondary education is associated with reduced risk of IPV for both women and their partners and that there is an increased protection when both women and their partners complete secondary education (Abramsky et al., 2011; Ackerson et al., 2008; Boyle et al., 2009; World Health Organization, 2012). Other predictors of IPV proffered by respondents in this study include poverty, “natural” tendency of men to be violent, negligence of roles in relationships, infidelity, denial of conjugal expectations, inter-tribal marriages, polygamy, interference from in-laws, westernization and gender equality campaigns leading to non-submission of women to their spouses. Research has demonstrated that causes of IPV are complex (Jewkes, 2002). In addition to male and female factors, equally important are contextual factors and ideologies—male superiority, culture of violence or environment permissive of violence (Jewkes, 2002; World Health Organization, 2012). These factors form a rich media on which IPV thrives. In a study conducted in rural South-East Nigeria, Ilika reported that community structures and sociocultural norms favour IPV, therefore, women excuse and condone acts of violence from their spouses (Ilika, 2005).

In this study, respondents described other triggers of IPV including alcohol use. In agreement with our findings, many researchers have described excessive consumption of alcohol as a potent predictor of violence among intimate partners (Foran & O’leary, 2008; Jewkes, 2002; Leonard, 2005; Wang et al., 2017; World Health Organization, 2012). According to Jewkes, alcohol accelerates the pathway to violence by lowering inhibitions, clouding judgment and making social cues nebulous (Jewkes, 2002). This assertion is also validated by the research findings of Caetano and colleagues (Caetano et al., 2001). Alcohol use has been reported to worsen outcomes of violence among intimate partners (Thompson & Kingree, 2006). However, causality and temporality is difficult to establish from research between alcohol use and IPV (Caetano et al., 2001).

IPV has physical, mental and social consequences not only for the victims or perpetrators but also for the whole family and the society as a whole. In consonance with our findings, the short and long-term consequences of IPV have been described in literature (Jewkes, 2002; Leonard, 2005; Ogbonnaya et al., 2019; Thompson & Kingree, 2006; World Health Organization, 2012). Consequences of IPV for the victims include chronic pain, gastrointestinal and gynaecological problems, increased HIV risk, anxiety, depression, substance abuse, low self-esteem, post-traumatic stress disorder and injury or death. (Anderson et al., 2003; Campbell et al., 2002; Karakurt et al., 2014; Kilpatrick, 2004). IPV against women during pregnancy can cause harm to both mothers and children (Campbell et al., 2002; A. O. A. O. Fawole et al., 2008). Self-reported personal consequences among men who perpetrate IPV include depression, guilty feelings, distraction at work and low self-esteem (Walker et al., 2010). Men reported anxiety and emotional distress such as worrying about what their abuse was doing to their children and concern about their partner leaving the relationship. The male perpetrators also reported their IPV behaviours to be negatively impacting their abilities at work and in relationships (Walker et al., 2010).

Participants also recognized that IPV has a negative effect on the children and the society. The adverse consequences that result from exposure of children to IPV include an increased risk of psychological, social, emotional and behavioural problems including mood and anxiety disorders, post-traumatic stress disorder, substance abuse and school-related problems among children and adolescents (Osofsky, 2009; Wathen & Macmillan, 2013). Such children are also more likely to have problems parenting and to maltreat their own children later in life as the negative effects may continue into adulthood and become part of an intergenerational cycle of violence (Osofsky, 2009; Schwartz et al., 2006). Children exposed to IPV are more likely to experience violent dating and intimate relationships either as victims or perpetrators in adulthood (Ehrensaft et al., 2003).

Preventive measures for IPV suggested by participants include spirituality, tolerance, patience, love, counselling, provision better housing for families, women empowerment. The role of spirituality in preventing and coping with IPV has been described (Bey, 2020; Howell et al., 2018; De la Rosa et al., 2016; Shaw et al., 2020). These suggested preventive strategies for IPV have some similarities and differences from what is recommended as best practices. The recommended best practices for preventing IPV include a need for comprehensive, multi-sectoral, long-term collaboration between government and civil society of ecological framework. They include teaching safe and healthy relationship skills, engaging influential adults and peers, disrupting the developmental pathways towards partner violence, creating protective environments, strengthening economic support for families and supporting survivors to increase safety and lessen harm (Niolon et al., 2017). Other preventive measures that had been described for both men and women include building the life-skills of young people through school-based programmes, early intervention services for families at risk of IPV including education and home visits, comprehensive support services for survivors, media and advocacy campaign to raise awareness about existing IPV legislation, community mobilization against IPV and women empowerment as well as legal reforms to protect women and vulnerable people (World Health Organization, 2012).

## Strengths and limitations

One of the strengths of this study is that the participants included leaders, professionals and policymakers in the civil service who are able to change societal norms. This study also provides unique insights into IPV and its effects on the population which is useful in planning community health services and interventions. However, the results of this study should be interpreted within the context of some limitations. First, qualitative studies are not designed to be generalizable, but rather are intended to improve the understanding of specific phenomena within a circumscribed sample; therefore, the theories and ideas can only be applied to other similar contexts. Our sample included men from different socio-economic and working classes who had at least primary education; therefore, findings of this study may not be applicable to the general population, particularly those with no formal education or extremes of socio-economic class. Secondly, social desirability bias cannot be ruled out as the respondents may have provided socially desirable responses given the sensitive nature of the topic. However, the respondents readily disclosed significant acts of violent behaviours that men perpetrate in intimate relationships particularly in a patriarchal society like Nigeria.

## Implications of this study

In order to end IPV, there is a need for preventive measures directed at all men and women, which are designed to balance power between men and women in intimate relationships. Most of the men were informed about IPV and the consequences but justified certain forms of IPV. This study revealed that men had misconceptions on masculinity which were culturally and socially supported and men stressed the importance of recognizing their power and control in intimate relationships. When men feel that these traits are not being recognized, they perceive their gender as being threatened.

There is a need for interventions that will promote respect and fairness between men and women. There is a need for policies that will train educational, health and social service providers, as well as to improve the legal system, while encouraging cultural awareness to promote social change. Participatory educational interventions should be used to encourage the values of respect and fairness. The interventions should aim at changing traditional gender roles rooted in patriarchal culture. There is also a need to increase awareness of men about their responsibilities and roles in promoting family well-being. Future programmes should implement educational interventions which encourage healthy relationships.

## Conclusion

IPV is a major public health problem in Nigeria. Men in the civil service expressed their views about the different forms of violence perpetrated and experienced in intimate relationships. The negative physical, mental and social consequences of IPV as it affects the victims and the perpetrators as well as their children and the society were discussed. There is a need for interventions targeted specifically at men to educate them on healthy relationships and the complications of IPV.

## Data Availability

The data that support the findings of this study are available on request from the corresponding author.

## Previous presentation

Part of this research findings with abstract number P123 was presented at the 20th Annual Women’s Health Congress held in Washington DC, USA in March, 2012.
